# Coexistence of quantum key distribution and optical communication with amplifiers over multicore fiber

**DOI:** 10.1515/nanoph-2023-0047

**Published:** 2023-04-24

**Authors:** Weiwen Kong, Yongmei Sun, Yaoxian Gao, Yuefeng Ji

**Affiliations:** The State Key Laboratory of Information Photonics and Optical Communications, School of Information and Communication Engineering, Beijing University of Posts and Telecommunications, Beijing 100876, China

**Keywords:** multicore fiber, noise analysis, quantum key distribution

## Abstract

In this paper, the influence of classical signals on quantum key distribution (QKD) is studied over multi-core fiber (MCF) when optical amplifiers exist. Firstly, the long-distance simultaneous transmission architectures of QKD and classical signals are proposed based on advanced asymmetric sending or not sending QKD (SNS-QKD) and classical Bennett–Brassard 1984-QKD (BB84-QKD), and the segment length between optical amplifiers can be adjusted according to requirement. Then, theoretical models of spontaneous Raman scattering noise and four-wave mixing noise are established based on the proposed architectures. Next, the calculation models of the secure key rate under the influence of noises from classical signals are derived. Finally, the experimental results show that the theoretical models match well with the experimental photons, and the maximum difference between experimental and simulated noise photons is less than 2.6 dB. Simulation results show that the performance of asymmetric SNS-QKD is better than that of BB84-QKD architecture when classical signals and quantum signals are transmitted in different cores of MCF.

## 1 Introduction

With the development of artificial intelligence and quantum computing, information security is a severe challenge in communication networks [1, 2]. Quantum key distribution (QKD) is based on the basic principles of quantum mechanics, combined with one-time pad technology to ensure the theoretical security of information [3, 4]. In recent years, QKD has made significant progress in extending secure transmission distances [5–8] and building large-scale networks [9–12]. For example, the proposal of twin-field QKD (TF-QKD) protocol brings hope for the long-distance transmission QKD [5]. In the classical Bennett–Brassard 1984-QKD (BB84-QKD) protocol, the secure key rate and channel transmittance *η* have a linear relationship, but in TF-QKD, the secure key rate and channel transmittance scale with $\sqrt{\eta }$ . This turning point development has brought opportunities for QKD in long-distance transmission. Several typical recording experiments were realized [10, 12, 13]. In [10, 12], the sending or not sending QKD (SNS-QKD) protocol belonging to the TF-QKD type is adopted, because the SNS-QKD protocol can tolerate a wider range of error rates caused by interference [11].

It is unrealistic to build a dedicated network for QKD, which requires a huge cost. Therefore, the integration of classical network and QKD network is an important development trend [14, 15]. The simultaneous transmission of quantum signals and classical signals in a single-core single-mode fiber (SSF) can expand the transmission capacity and reduce the cost of fiber deployment, but it also brings some problems. The power of the classical signals per channel is typically 0 dBm, and the power of the quantum signals is usually lower than −80 dBm [16, 17]. Therefore, the weak quantum signals will be interfered by the noises generated from the classical signals, such as spontaneous Raman scattering (SpRS) noise and four-wave mixing (FWM) noise [18, 19]. The SpRS noise spectrum exceeds 200 nm, and FWM noise appears on a specific channel, so these noises can easily fall on the quantum channels. At present, some measures to suppress SpRS noise and FWM noise on the quantum channels have been proposed, such as reducing the power of classical signals [19, 20], time-domain filtering [21, 22], and frequency-domain filtering [22]. In addition, some effective channel allocation schemes have been proposed, which can effectively suppress the noises on the quantum channels [23–27].

However, with the improvement of data requirements, the existing SSF is gradually approaching its capacity limit, and it is very difficult to further increase the fiber capacity [28, 29]. In order to meet the demand for large-capacity services in the future, space division multiplexing (SDM) technology is proposed. Typical fiber types that implement SDM include multi-mode fibers and multi-core fibers (MCF) [30–33], among them; MCF has more room for capacity improvement. Compared with multiple SSFs, MCF has high space efficiency [34]. In other words, MCF can provide higher capacity in the same space, and can be applied in some scenarios, such as submarine optical communications that require a high number of spatial channels and data centers with tight fiber port space [35, 36]. Therefore, research on MCF starts gradually in recent years. Although the cost of MCF is currently high, the cost is expected to decrease with the advancement of technology and the increase of demand.

Furthermore, the high-dimensional encoding of quantum states on MCF [37, 38] and the simultaneous transmission with classical signals are also gradually being studied. The simultaneous transmission of quantum signals and classical signals in a MCF will not only be affected by the SpRS noise and FWM noise existing in the same core, but also by the inter-core crosstalk (ICXT) noise between cores of MCF [34, 39, 40]. In addition, the SpRS noise and FWM noises will also generate inter-core SpRS (ICSpRS) noise and inter-core FWM (ICFWM) noise in other cores [41–44]. In [45], the transmission experiment of classical signals and QKD in MCF was demonstrated for the first time, and the secure key rate of 605 kbps was achieved over a 53 km 7-core fiber. In [17], the secure key rate of 105.7 Mbps was achieved over a 7.9 km 37-core fiber, which increased the transmission capacity of QKD and data signals. Then, our team adopted the wavelength interleaving scheme of classical signals and quantum signals, and realized the simultaneous transmission experiment over 1 km 7-core fiber, and the secure key rate can reach up to 10 kbps [46]. Subsequently, a recorded classical data rate of 11.2 Tbps and QKD was simultaneously transmitted experimentally in a 1 km 7-core fiber, and the maximum secure key rate can reach 1.4 kbps [47]. Recently, in [48], QKD and 25 dBm high-power classical signals transmitted simultaneously over 10 km 7-core MCF, ICSpRS noise was analyzed theoretically and considered experimentally. In addition, for the impact of ICSpRS and ICFWM noises on QKD, ICSpRS noise was simulated and measured in [41, 45], and theoretically modeled in [42]. Furthermore, the ICSpRS and ICFWM noises are theoretically modeled in [43].

For the previous research, most of them are based on the simultaneous transmission of BB84-QKD, and the transmission distance is generally not more than 120 km, and the classical signals cannot need to pass through repeater. For long-distance QKD transmission, such as TF-QKD, the secure transmission distance is usually more than 120 km. The classical signals need to be allocated an amplifier every 80 km ∼ 120 km [49, 50]. Therefore, the theoretical models of intra-core noises and inter-core noises without classical amplifier are no longer applicable in the MCF where the classical signals and QKD are transmitted together.

In this paper, for the long-distance simultaneous transmission of QKD and classical signals, we propose simultaneous transmission architectures based on asymmetric SNS-QKD and BB84-QKD. In the architectures, optical amplifiers are allocated in the each segment of classical link. In asymmetric SNS-QKD architecture, the distance between Alice and Charlie and the distance between Bob and Charlie can be different, and the length of each segment can be unequal in asymmetric SNS-QKD and BB84-QKD architecture. Also, we establish theoretical models of SpRS noise and FWM noise based on the proposed simultaneous transmission architectures, which include intra-core noises, inter-core noises, forward noises, and backward noises. Then, the secure key rate with asymmetric SNS-QKD under the influence of classical signals is derived. Finally, simulation and experiment are performed. When there is no noise, the asymmetric SNS-QKD simultaneous transmission architecture extends the secure transmission distance by 255 km compared to the BB84-QKD architecture, showing the advantages of TF-QKD. However, the transmission distance drops significantly for the asymmetric SNS-QKD architecture compared to the BB84-QKD when intra-core noises are present. The experimental results show that the proposed theoretical model matches the experimental data well.

## 2 Simultaneous transmission architectures and noise theoretical models

In the current research on discrete variable QKD, there are mainly two types of QKD architectures, one is the Alice–Charlie–Bob architecture represented by the measurement-device-independent QKD (MDI-QKD) and TF-QKD, and the other is the Alice–Bob architecture represented by the BB84-QKD. When the distance of classical transmission exceeds 80 km ∼ 120 km, it generally needs to add classical relays, which will affect the performance of QKD. Therefore, we consider the influence of the noises generated from classical signals on the performance of QKD when classical relay nodes exist, including insertion loss and optical amplifier in the nodes. TF-QKD is discussed with the asymmetric SNS-QKD protocol [51] because of its high tolerance for fiber attenuation and asymmetry between Alice–Charlie and Bob–Charlie, and the following analysis is carried out separately.

### 2.1 Asymmetric SNS-QKD

**Figure 1:**
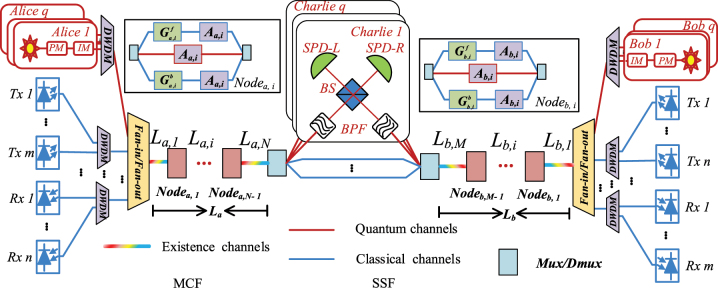
Asymmetric SNS-QKD architecture of simultaneous transmission with classical and quantum signals (PM, phase modulator; IM, intensity modulator; DWDM, dense wavelength division multiplexer; Tx, transmitter of classical signals; Rx, receiver of classical signals; *L*_*a*,*i*_ (*L*_*b*,*i*_), the length of *i*-th segment from Alice (Bob) to Charlie; *A*_*a*,*i*_(*A*_*b*,*i*_), the insertion attenuation of *i*-th node from Alice (Bob) to Charlie; $G_{a,i}^{f}$ ( $G_{b,i}^{f}$ ), the amplifier gain of the forward classical signals in the *i*-th segment from Alice (Bob) to Charlie; $G_{a,i}^{b}$ ( $G_{b,i}^{b}$ ), the amplifier gain of the backward classical signals in the *i*-th segment from Alice (Bob) to Charlie; *L*_*a*_(*L*_*b*_), the length of Alice (Bob) to Charlie; SPD, single photon detector; BS, beam splitter; BPF, band pass filter).

Figure 1 shows the simultaneous transmission architecture of asymmetric SNS-QKD and classical signals, with classical relays in the architecture. The lengths of *L*_*a*_ and *L*_*b*_ can be adjusted according to deployment requirements. *L*_*a*_ and *L*_*b*_ are divided into *N* segments and *M* segments, respectively by the classical relays, and the length of each segment can also be adjusted. The *q* quantum wavelengths are sent from Alice and Bob, and received at Charlie. Classical signals are bi-directional transmission. We define *m* classical wavelengths from Alice to Bob as forward signals, and *n* classical wavelengths from Bob to Alice as backward signals. For each node, separate the classical forward signals, the classical backward signals, and the quantum signals, then amplify the classical signals before combining in nodes.

In the architecture of Figure 1, the quantum signals in each segment of MCF will be affected by the noises generated from the classical signals. This paper mainly considers FWM noise and SpRS noise, including the corresponding intra-core noises, inter-core noises, forward noises, and backward noises. The power of forward intra-core FWM noise (F-FWM) can be represented as [24]:

(1) $$\begin{array}{ll} P_{F-FWM}\left(L\right) & =\frac{\eta^{\prime }D^{2}\gamma^{2}P_{i}P_{j}P_{k}\exp\left(-\alpha L\right)}{9\alpha^{2}}\\ & \;\times {\left[1-\exp\left(-\alpha L\right)\right]}^{2}. \end{array}$$

*D* is degeneracy factor of FWM. *γ* is nonlinearity coefficient. *P*_*i*_ *P*_*j*_ *P*_*k*_ are the power of arbitrary three classical pump signals. *α* and *L* are the fiber attenuation and length, respectively. *η*′ is the FWM efficiency, and can be expressed as [24]:

(2) $$\eta^{\prime }=\frac{\alpha^{2}}{\alpha^{2}+\Delta \beta^{2}}\left(1+\frac{4\exp\left(-\alpha L\right)\sin^{2}\left(\frac{\Delta \beta L}{2}\right)}{{\left[1-\exp\left(-\alpha L\right)\right]}^{2}}\right).$$

Δ*β* is the phase matching factor. Based on the Equation (1), the F-FWM noise on the quantum channels in the architecture of Figure 1 can be calculated. The main idea is to calculate in segments, and then add the noise that reaches Charlie in each segment. The calculation method from Alice to Charlie is the same as the calculation method from Bob to Charlie, so Alice to Charlie is used as an example for modeling.

The F-FWM noise on the quantum channels in the first segment of Alice to Charlie can be represented as:

(3) $$\begin{array}{ll} P_{F-FWM,seg1}^{a,q} & =P_{F-FWM}^{L_{a,1}}\exp\left(-\alpha_{q}\cdot \overset{N}{\underset{i=2}{\sum }}\left(L_{a,i}\right)\right)\cdot \overset{N}{\underset{i=1}{\prod }}A_{a,i}. \end{array}$$

$P_{F-FWM}^{L_{a,1}}$ represents the power of F-FWM noise in the *L*_*a*,1_. *L*_*a*,*i*_ is the length of *i*-th segment from Alice to Charlie. *α*_*q*_ is the attenuation on the quantum channels. *A*_*a*,*i*_ is the insertion attenuation of *i*-th node from Alice to Charlie. For convenience, define the attenuation of Mux/Dmux as *A*_*a*,*N*_.

The pump power of F-FWM noise on the second segment should consider the attenuation of first segment and Node_*a*,1_, and the amplification of classical signals in Node_*a*,1_. After that, F-FWM noise is generated on the second segment, and then attenuated at the following segments and nodes, reaching Charlie. Therefore, it can be represented as:

(4) $$\begin{array}{ll} P_{F-FWM,seg2}^{a,q} & =P_{F-FWM}^{L_{a,2}}\exp\left(-\alpha_{q}\cdot \overset{N}{\underset{i=3}{\sum }}\left(L_{a,i}\right)\right)\\ & \;\cdot \overset{N}{\underset{i=2}{\prod }}A_{a,i}. \end{array}$$

The F-FWM noise of *N* segments from Alice to Charlie is calculated and then added. After reaching Charlie, it can be expressed as:

(5) $$\begin{array}{ll} P_{F-FWM}^{a,q} & =\overset{N}{\underset{j=1}{\sum }}\left[P_{F-FWM}^{L_{a,j}}\exp\left(-\alpha_{q}\cdot \overset{N}{\underset{i=j+1}{\sum }}\left(L_{a,i}\right)\right)\right.\\ & \;\left.\cdot \overset{N}{\underset{i=j}{\prod }}A_{a,i}\right]. \end{array}$$

*j* represents the *j*-th segment. The backward intra-core FWM (B-FWM) noise is studied, and it is generated by the backward Rayleigh scattering of F-FWM noise. Therefore, the noise of B-FWM can be calculated by Equation (6).

(6) $$P_{B-FWM}\left(L\right)=S\alpha_{R}\overset{L}{\underset{0}{\int }}P_{F-FWM}\left(l\right)\exp\left[-\alpha \left(l\right)\right]dl.$$

*L* is transmission distance. *S* represents the recapture factor of the Rayleigh scattering component into the backward direction, and *α*_*R*_ denotes the attenuation coefficient of Rayleigh scattering. The pump light of B-FWM noise is backward classical signals from Bob to Alice. The B-FWM noise on the quantum channels in the *N*-th segment of Alice to Charlie can be represented as:

(7) $$P_{B-FWM,segN}^{a,q}=P_{B-FWM}^{L_{a,N}}A_{a,N}.$$

$P_{B-FWM}^{L_{a,N}}$ represents the power of B-FWM noise in the *L*_*a*,*N*_. The pump power of B-FWM noise on the *N* − 1-th segment should consider the attenuation of *N*-th segment and Node_*a*,*N*−1_, and the amplification of classical signals in node Node_*a*,*N*−1_. After that, B-FWM noise is generated on the *N* − 1-th segment, and then attenuated at Node_*a*,*N*−1_ and *N*-th segments, reaching Charlie. Therefore, it can be represented as:

(8) $$\begin{array}{ll} P_{B-FWM,segN-1}^{a,q} & =P_{B-FWM}^{L_{a,N-1}}\cdot A_{a,N-1}\\ & \;\times \exp\left(-\alpha_{q}\cdot L_{a,N}\right)A_{a,N}. \end{array}$$

The B-FWM noise of *N* segments from Alice to Charlie is calculated and then added. After reaching Charlie, it can be expressed as:

(9) $$\begin{array}{ll} P_{B-FWM}^{a,q} & =\overset{N}{\underset{j=1}{\sum }}\left\{P_{B-FWM}^{L_{a,j}}\overset{N-1}{\underset{i=j}{\prod }}\right.\\ & \;\left.\times \left[A_{a,i}\cdot \exp\left(-\alpha_{q}\cdot L_{a,i+1}\right)\right]\right\}\cdot A_{a,N}. \end{array}$$

For forward intra-core SpRS noise (F-SpRS), the power of F-SpRS noise within a bandwidth of Δ*λ* can be represented as the Equation (10) [52].

(10) $$P_{F-SpRS}\left(L\right)=P\cdot \exp\left(-\alpha L\right)\cdot L\cdot \rho \left(\lambda_{c},\lambda_{q}\right)\cdot \Delta \lambda .$$

*λ*_*c*_ is the wavelength of classical signal, *λ*_*q*_ is the wavelength of quantum signal. $\rho \left(\lambda_{c},\lambda_{q}\right)$ denotes the normalized SpRS cross-section (per fiber length and bandwidth), and it can be obtained from [19]. The F-SpRS noise of *N* segments from Alice to Charlie is calculated and then added. After reaching Charlie, it can be expressed as:

(11) $$\begin{array}{ll} P_{F-SpRS}^{a,q} & =\overset{N}{\underset{j=1}{\sum }}\left[P_{F-SpRS}^{L_{a,j}}\exp\left(-\alpha_{q}\cdot \overset{N}{\underset{i=j+1}{\sum }}\left(L_{a,i}\right)\right)\right.\\ & \;\left.\cdot \overset{N}{\underset{i=j}{\prod }}A_{a,i}\right]. \end{array}$$

$P_{F-SpRS}^{L_{a,j}}$ represents the power of F-SpRS noise in *L*_*a*,*j*_. The backward intra-core SpRS (B-SpRS) can be calculated by Equation (12) [52].

(12) $$P_{B-SpRS}\left(L\right)=P\cdot \frac{\left[1-\exp\left(-2\alpha L\right)\right]}{2\alpha }\cdot \rho \left(\lambda_{c},\lambda_{q}\right)\cdot \Delta \lambda .$$

The B-SpRS noise of *N* segments from Alice to Charlie is calculated and then added. After reaching Charlie, it can be expressed as:

(13) $$\begin{array}{ll} P_{B-SpRS}^{a,q} & =\overset{N}{\underset{j=1}{\sum }}\left\{P_{B-SpRS}^{L_{a,j}}\overset{N-1}{\underset{i=j}{\prod }}\right.\\ & \;\left.\times \left[A_{a,i}\cdot \exp\left(-\alpha_{q}\cdot L_{a,i+1}\right)\right]\right\}\cdot A_{a,N}. \end{array}$$

$P_{B-SpRS}^{L_{a,j}}$ represents the power of B-SpRS noise in the *L*_*a*,*j*_. For other noises in Figure 1, such as F-ICFWM noise, B-ICFWM noise, F-ICSpRS noise, and B-ICSpRS noise, these noise models without classical amplifiers are proposed in [43]. In the asymmetric SNS-QKD architecture where classical amplifiers exist, they are also calculated according to the segment, and the method is consistent with the above-mentioned intra-core noise model. Please refer to Appendix A for the calculation method.

### 2.2 BB84-QKD

**Figure 2:**
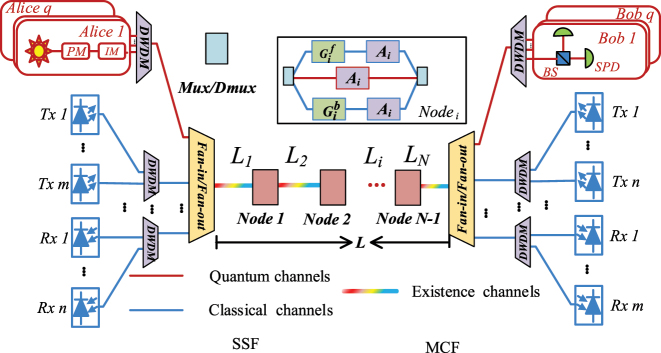
BB84-QKD architecture with simultaneous transmission of classical and quantum signals (*L*_*i*_, the length of *i*-th segment from Alice to Bob; *A*_*i*_, the insertion loss of *i*-th node; $G_{i}^{f}$ , the amplifier gain of the forward classical signals in node *i* from Alice to Bob; $G_{i}^{b}$ , the amplifier gain of the backward classical signals in node *i* from Bob to Alice; *L*, the length of Alice to Bob).

In the BB84-QKD-based quantum signals and classical signals transmission architecture as shown in Figure 2, the quantum signals go from Alice to Bob. The classical forward signals and the quantum signals are in the same direction, that is, from Alice to Bob, and the classical backward signals and the quantum signals are reversed, that is, from Bob to Alice. The node model is the same as the asymmetric SNS-QKD-based transmission architecture. The distance between Alice and Bob is *L*, which is divided into *N* segments by the classical relays, and each segment has arbitrary length. For convenience, define the attenuation of fan-in/fan-out and DWDM before Bob as *A*_*N*_.

For F-FWM noise, the noise introduced from the classical forward signals. By calculating the noise on each segment and considering the attenuation and amplification of the classical relays, the F-FWM noise on the quantum channels reaching Charlie can be expressed as:

(14) $$\begin{array}{ll} P_{F-FWM}^{q} & =\overset{N}{\underset{j=1}{\sum }}\left[P_{F-FWM}^{L_{j}}\exp\left(-\alpha_{q}\cdot \overset{N}{\underset{i=j+1}{\sum }}\left(L_{i}\right)\right)\right.\;\left.\cdot \overset{N}{\underset{i=j}{\prod }}A_{i}\right]. \end{array}$$

The B-FWM noise is generated from the classical backward signals, and the B-FWM noise of *N* segments on the quantum channels from Alice to Bob is calculated and then added. After reaching Charlie, it can be expressed as:

(15) $$\begin{array}{ll} P_{B-FWM}^{q} & =\overset{N}{\underset{j=1}{\sum }}\left\{P_{B-FWM}^{L_{j}}\overset{N-1}{\underset{i=j}{\prod }}\right.\\ & \;\left.\times \left[A_{i}\cdot \exp\left(-\alpha_{q}L_{i+1}\right)\right]\right\}A_{N}. \end{array}$$

For F-SpRS noise and B-SpRS noise, the modeling idea is the same as that of F-FWM noise and B-FWM noise. After segmented calculation, add them together, as shown in Equations (16) and (17).

(16) $$\begin{array}{ll} P_{F-SpRS}^{q} & =\overset{N}{\underset{j=1}{\sum }}\left[P_{F-SpRS}^{L_{j}}\exp\left(-\alpha_{q}\cdot \overset{N}{\underset{i=j+1}{\sum }}\left(L_{i}\right)\right)\right.\\ & \;\left.\cdot \overset{N}{\underset{i=j}{\prod }}A_{i}\right]. \end{array}$$

(17) $$\begin{array}{ll} P_{B-SpRS}^{q} & =\overset{N}{\underset{j=1}{\sum }}\left\{P_{B-SpRS}^{L_{j}}\overset{N-1}{\underset{i=j}{\prod }}\right.\\ & \;\left.\times \left[A_{i}\cdot \exp\left(-\alpha_{q}L_{i+1}\right)\right]\right\}A_{N}. \end{array}$$

For inter-core noises, the models in the BB84-QKD-based simultaneous transmission architecture of quantum signals and classical signals are the same calculation method as intra-core noise. Please refer to Appendix B for the calculation method.

## 3 Secure key rate of asymmetric SNS-QKD and BB84-QKD with classical signals

When the quantum signals and the classical signals are transmitted together, the calculation method of the secure key rate needs to be derived, because the noises generated from the classical signals need to be considered in the theoretical models. Therefore, the following are the theoretical derivations for calculation the secure key rate of asymmetric SNS-QKD and BB84-QKD when classical signals coexist.

### 3.1 Decoy-state asymmetric SNS-QKD

In asymmetric SNS-QKD, Alice and Bob randomly choose the Z window and the X window, the Z window is signal window and the X window is decoy window. In Z window, Alice (Bob) determines to send a signal state pulse $\left|\sqrt{\mu_{a}}{\text{e}}^{i\delta_{a}+i\gamma_{a}}\right\rangle$ $\left(\left|\sqrt{\mu_{b}}{\text{e}}^{i\delta_{b}+i\gamma_{b}}\right\rangle \right)$ with probability *ϵ*_*a*_ (*ϵ*_*b*_), and not to send with 1 − *ϵ*_*a*_ (1 − *ϵ*_*b*_). *μ*_*a*_ and *μ*_*b*_ denote signal intensities that Alice and Bob send pulses, respectively. *δ*_*a*_ and *δ*_*b*_ denote random phases that Alice and Bob send pulses, respectively. *γ*_*a*_ and *γ*_*b*_ are the global phases. In X window, Alice and Bob emit decoy-state pulses $\left|\sqrt{\alpha }{\text{e}}^{i\delta_{a}+i\gamma_{a}}\right\rangle$ and $\left|\sqrt{\beta }{\text{e}}^{i\delta_{a}+i\gamma_{a}}\right\rangle$ , respectively. $\alpha \in \left\{\upsilon_{a},\omega_{a},o\right\}$ , and $\beta \in \left\{\upsilon_{b},\omega_{b},o\right\}$ . *υ*_*a*_ > *ω*_*a*_, *υ*_*b*_ > *ω*_*b*_. *υ*_*a*_, *ω*_*a*_, *υ*_*b*_ and *ω*_*b*_ are the decoy-state intensities of Alice and Bob. *o* represents the vacuum sources [51].

Supposed that Alice and Bob send pulses with intensities *x*_*a*_, *x*_*b*_, respectively, and corresponding transmittances are *η*_*a*_, *η*_*b*_ (*η*_*a*_ > *η*_*b*_). For simplicity, we assume that the two detectors on both sides of Charlie are the same, and each with a dark count rate *p*_*d*_, noise count rate from classical signals *p*_*c*_, and detection efficiency *η*_*d*_ [51].

The counting rate of the *n*-photon states which causes effective events can be represented as:

(18) $$\begin{array}{ll} Q_{n}^{x_{a}x_{b}} & =\overset{n}{\underset{m=0}{\sum }}\frac{e^{-x_{a}}x_{a}^{m}}{m!}\frac{e^{-x_{b}}x_{b}^{n-m}}{\left(n-m\right)!}\\ & \;\times \left[1-{\left(1-p_{d}-p_{c}\right)}^{2}{\left(1-\eta_{a}\right)}^{m}{\left(1-\eta_{b}\right)}^{n-m}\right]. \end{array}$$

(19) $$\;p_{c}=\frac{\left(P_{\text{sum}}^{A-C}+P_{\text{sum}}^{B-C}\right)\cdot \eta_{d}\cdot \tau_{\text{gate}}}{2\times h\times f}.$$

$P_{\text{sum}}^{A-C}$ and $P_{\text{sum}}^{B-C}$ are the noise power generated by classical signals from Alice to Charlie and the noise power from Bob to Charlie. *τ*_gate_ is the gate width of detector, and *h* is the Planck’s constant (6.63 × 10^−34^ J s). *f* is the frequency of quantum signal. The noise power $P_{\text{sum}}^{A-C}$ or $P_{\text{sum}}^{B-C}$ should be the noises as follow:

(20) $$\begin{array}{ll} P_{\text{sum}}^{X-C} & =P_{F-SpRS}^{X-C}+P_{B-SpRS}^{X-C}+P_{F-FWM}^{X-C}+P_{B-FWM}^{X-C}\\ & \;+P_{F-ICSpRS}^{X-C}+P_{B-ICSpRS}^{X-C}\\ & \;+P_{F-ICFWM}^{X-C}+P_{B-ICFWM}^{X-C}. \end{array}$$

Equation (18) is called the *n*-photon effective event. When there are *m* photons from Bob and *n* − *m* photons from Alice, the equivalent photon number distribution is as follow [51]:

(21) $$P_{n}^{\prime }\left(x_{a}+x_{b}\right)=\overset{n}{\underset{m=0}{\sum }}\frac{e^{-x_{a}}x_{a}^{m}}{m!}\frac{e^{-x_{b}}x_{b}^{n-m}}{\left(n-m\right)!}.$$

Then, the equivalent yield of the *n*-photon effective event can be formulated as:

(22) $$\begin{array}{ll} Y_{n}^{x_{a}x_{b}} & =\frac{Q_{n}^{x_{a}x_{b}}}{P_{n}^{\prime }\left(x_{a}+x_{b}\right)}\\ & =1-{\left(1-p_{d}-p_{c}\right)}^{2}{\left[\frac{\frac{x_{a}}{x_{b}}\left(1-\eta_{a}\right)+\left(1-\eta_{b}\right)}{\frac{x_{a}}{x_{b}}+1}\right]}^{n}. \end{array}$$

For convenience, denote $Y_{n}^{x_{a}x_{b}}$ as $Y_{n}^{k}$ , and $Q_{n}^{x_{a}x_{b}}$ as $Q_{n}^{k}$ . In addition, *ω*_*a*_ + *ω*_*b*_ = *μ*_1_, *υ*_*a*_ + *υ*_*b*_ = *μ*_2_. $\frac{\omega_{a}}{\omega_{b}}=k_{1}$ , $\frac{\upsilon_{a}}{\upsilon_{b}}=k_{2}$ . For *k*_1_ ≤ *k*_2_, the lower bound of single-photon yield in X window can be get [51]:

(23) $$\begin{array}{ll} Y_{1}^{L} & =\frac{P_{2}^{\prime }\left(\mu_{2}\right)Q_{\mu_{1}}-P_{2}^{\prime }\left(\mu_{1}\right)Q_{\mu_{2}}}{P_{2}^{\prime }\left(\mu_{2}\right)P_{1}^{\prime }\left(\mu_{1}\right)-P_{2}^{\prime }\left(\mu_{1}\right)P_{1}^{\prime }\left(\mu_{2}\right)}\\ & \;+\frac{\left[P_{2}^{\prime }\left(\mu_{1}\right)P_{0}^{\prime }\left(\mu_{2}\right)-P_{2}^{\prime }\left(\mu_{2}\right)P_{0}^{\prime }\left(\mu_{1}\right)\right]Y_{0}}{P_{2}^{\prime }\left(\mu_{2}\right)P_{1}^{\prime }\left(\mu_{1}\right)-P_{2}^{\prime }\left(\mu_{1}\right)P_{1}^{\prime }\left(\mu_{2}\right)}. \end{array}$$

Next, define $\frac{\mu_{1}}{\mu_{2}}\geq k_{1}$ to estimate the single-photon yield of the Z window, and the yield of the X window is not greater than the yield of the Z window. Therefore, $Y_{1}^{L}$ is considered the lower bound of the Z window. The quantum bit error rate (QBER) of a single photon can be expressed as [7]:

(24) $$e_{1}\leq e_{1}^{U}=\frac{Q_{\mu_{1}}E_{\mu_{1}}-P_{0}^{\prime }\left(\mu_{1}\right)Y_{0}e_{0}}{P_{1}^{\prime }\left(\mu_{1}\right)Y_{1}^{L}}.$$

*e*_0_ is 0.5. In actual experiments, the average count rate and QBER of the X window can be directly measured. This paper uses the linear model in [51] to estimate the result. A two-mode state $\left|\sqrt{\alpha }{\text{e}}^{i\delta_{a}}\right\rangle \;\left|\sqrt{\beta }{\text{e}}^{i\delta_{b}}\right\rangle$ goes through the quantum channels and a beam-splitter, and it turns into $\left|\sqrt{\frac{\alpha \eta_{a}}{2}}{\text{e}}^{i\delta_{a}}+\sqrt{\frac{\beta \eta_{b}}{2}}{\text{e}}^{i\delta_{b}}\right\rangle \;⊗\;\left|\sqrt{\frac{\alpha \eta_{a}}{2}}{\text{e}}^{i\delta_{a}}-\sqrt{\frac{\beta \eta_{b}}{2}}{\text{e}}^{i\delta_{b}}\right\rangle$ . The corresponding gains $\left(Q_{\alpha \beta }^{\delta_{a}\delta_{b}}\right)$ and the QBER $\left(Q_{\alpha \beta }^{\delta_{a}\delta_{b}}E_{\alpha \beta }^{\delta_{a}\delta_{b}}\right)$ are given by:

(25) $$\begin{array}{ll} Q_{\alpha \beta }^{\delta_{a}\delta_{b}} & =\left(1-p_{d}-p_{c}\right)e^{-\frac{\alpha \eta_{a}}{2}-\frac{\beta \eta_{b}}{2}}\left(e^{-\cos\left(\delta_{a}-\delta_{b}\right)\sqrt{\alpha \beta \eta_{a}\eta_{b}}}\right.\\ & \;\left.+e^{\cos\left(\delta_{a}-\delta_{b}\right)\sqrt{\alpha \beta \eta_{a}\eta_{b}}}\right)\\ & \;-2{\left(1-p_{d}-p_{c}\right)}^{2}{\text{e}}^{-\alpha \eta_{a}-\beta \eta_{b}}. \end{array}$$

(26) $$\begin{array}{ll} Q_{\alpha \beta }^{\delta_{a}\delta_{b}}E_{\alpha \beta }^{\delta_{a}\delta_{b}} & =\left(1-p_{d}-p_{c}\right)e^{-\frac{\alpha \eta_{a}}{2}-\frac{\beta \eta_{b}}{2}-\cos\left(\delta_{a}-\delta_{b}\right)\sqrt{\alpha \beta \eta_{a}\eta_{b}}}\\ & \;-{\left(1-p_{d}-p_{c}\right)}^{2}{\text{e}}^{-\alpha \eta_{a}-\beta \eta_{b}}. \end{array}$$

The range of $\left|\delta_{a}-\delta_{b}\right|$ is $\left[0,\frac{2\pi }{M}\right]\cup \left[\pi ,\pi +\frac{2\pi }{M}\right]$ when the post-processing of the X window is executed. M is the number of phase slices predetermined by users. Defined that the misalignment error of QKD system is *E*_*d*_, and the system error rate can be represented as [51]:

(27) $$E_{s}=\frac{a}{2}-\frac{\sqrt{x_{a}\eta_{a}x_{b}\eta_{b}}}{x_{a}\eta_{a}+x_{b}\eta_{b}}+\frac{b\sqrt{x_{a}\eta_{a}x_{b}\eta_{b}}}{x_{a}\eta_{a}+x_{b}\eta_{b}}E_{d}.$$

However, there will be an additional phase difference between Alice and Bob during the single-photon interference process, which can be expressed as *U* = arccos(1 − 2*E*_*s*_). The average gain and QBER can be expressed as:

(28) $$Q_{\alpha \beta }=\frac{M^{2}}{4\pi^{2}}\overset{\frac{2\pi }{M}+U}{\underset{U}{\int }}\overset{\frac{2\pi }{M}}{\underset{0}{\int }}Q_{\alpha \beta }^{\delta_{a}\delta_{b}}d\delta_{a}d\delta_{b}.$$

(29) $$Q_{\alpha \beta }E_{\alpha \beta }=\frac{M^{2}}{4\pi^{2}}\overset{\frac{2\pi }{M}+U}{\underset{U}{\int }}\overset{\frac{2\pi }{M}}{\underset{0}{\int }}Q_{\alpha \beta }^{\delta_{a}\delta_{b}}E_{\alpha \beta }^{\delta_{a}\delta_{b}}d\delta_{a}d\delta_{b}.$$

Finally, with the above formulas, the secure key rate can be expressed as [51]:

(30) $$\begin{array}{ll} R_{\text{pulse}} & =P_{za}P_{zb}\left\{\left[\epsilon_{a}\left(1-\epsilon_{b}\right)e^{-u_{a}}u_{a}+\epsilon_{b}\left(1-\epsilon_{a}\right)e^{-u_{b}}u_{b}\right]\right.\\ & \;\left.\cdot Y_{1}^{L}\times \left[1-H\left(e_{1}^{U}\right)\right]-Q_{u_{a}u_{b}}f^{\prime }H\left(E_{u_{a}u_{b}}\right)\right\}. \end{array}$$

The probability that Alice randomly selects the Z window is *P*_*za*_, and the probability that Bob randomly selects the Z window is *P*_*zb*_. $Q_{u_{a}u_{b}}$ and $E_{u_{a}u_{b}}$ are the average gain and QBER of effective events in Z window; *f*′ is the error correction efficiency. *H*(⋅) is the binary Shannon entropy.

### 3.2 Decoy-state BB84-QKD

The decoy state BB84-QKD can solve the photon number splitting attack caused by multiple photons per pulse, so it is widely used in research. The dark count noise and intra-core and inter-core noises should be considered, and are expressed as under 2 SPDs phase-encoded QKD:

(31) $$\begin{array}{ll} P_{\text{sum}} & =2P_{\text{dark}}+P_{F-SpRS}+P_{B-SpRS}+P_{F-FWM}\\ & \;+P_{B-FWM}+P_{F-ICSpRS}\\ & \;+P_{B-ICSpRS}+P_{F-ICFWM}+P_{B-ICFWM}. \end{array}$$

The total noise photon probability *Y*_0_ can be expressed as:

(32) $$Y_{0}=\frac{P_{\text{sum}}\cdot \eta_{d}\cdot \tau_{\text{gate}}}{2h\cdot f}$$

*P*_sum_ is the noise power generated by classical signals from Alice to Bob. *η*_*d*_ is the efficiency of detector. *τ*_gate_ is the gate width of detector, and *h* is the Planck’s constant. *f* is the frequency of quantum signal. Supposed that *Q*_1_ is the single photon gain, *E*_1_ is the QBER caused by the single photon state, *Q*_*μ*_ is the gain of the signal state, and *E*_*μ*_ is the QBER of the signal state, and they can be represented as:

(33) $$\left\{\begin{array}{cc} Q_{1} & =\left(Y_{0}+\eta \right)\cdot \mu \cdot e^{-\mu },\\ E_{1} & =\frac{1}{Y_{1}}\cdot \left(\frac{1}{2}Y_{0}+e_{\text{Det}}\eta \right),\\ Q_{\mu } & =Y_{0}+1-{\text{e}}^{-\eta \mu },\\ E_{\mu } & =\frac{1}{Q_{\mu }}\left[\frac{1}{2}Y_{0}+e_{\text{Det}}\left(1-{\text{e}}^{-\eta \mu }\right)\right],\\ Y_{1} & =Y_{0}+\eta . \end{array}\right.$$

*η* is the efficiency at which the photons sent by Alice are detected by Bob. *e*_Det_ is the probability of error detection results caused by signal light, and the magnitude depends on fringe visibility. The lower bound of the secure key rate *R*_pulse_ under the decoy-state BB84 can be expressed as [53]:

(34) $$R_{\text{pulse}}\geq q\left\{Q_{1}\left[1-H_{2}\left(E_{1}\right)\right]-Q_{\mu }f^{\prime }H_{2}\left(E_{\mu }\right)\right\}.$$

*q* is the protocol-related efficiency, which is 0.5 in the BB84 protocol.

## 4 Simulations and results

This section is mainly divided into two parts. First, simulate and analyze the noises in the asymmetric SNS-QKD and BB84-QKD architectures, and then analyze the secure key rate based on the asymmetric SNS-QKD and BB84-QKD architectures. Simulation parameters are shown in the Table 1. Phase-encoded QKD is used in the simulation. The amplifier gain at each node compensates for the attenuation of the previous segment of fiber and node, so the amplified classical signal power is fixed for any node.

**Table 1:** Simulation parameters with asymmetric SNS-QKD and BB84-QKD.

Parameters	Values	Parameters	Values
*A*_*a*,*i*_, *A*_*b*,*i*_, *A*_*i*_	2 dB	Coupling coefficient of MCF	10^−5^/km
Fiber loss	0.21 dB/km	Nonlinear coefficient	1.3 W/km
Dispersion slope	0.056 ps/nm^2^/km	Dispersion constant	17 ps/nm/km
Phase slice for asymmetric SNS-QKD	16	Average number of photons per pulse	0.4
Misalignment error probability	1.2 %	Error correction efficiency	1.15
Detector efficiency	15 %	Detection probability of dark counts	10^–9^

### 4.1 Noise analysis

**Figure 3:**
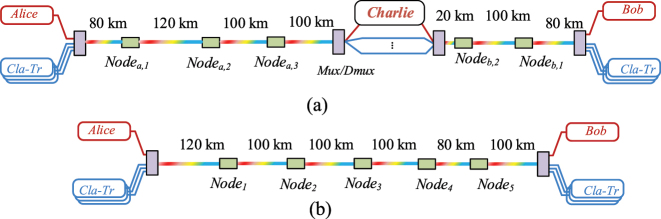
Simulation architecture. (a) Asymmetric SNS-QKD architecture with simultaneous transmission of classical and quantum signals. (b) BB84-QKD architecture with simultaneous transmission of classical and quantum signals.

Simultaneous transmission asymmetric SNS-QKD architecture in the simulation is shown in Figure 3(a). The distance between Alice and Bob is 600 km, and there are a total of 5 classical relays. The distance from Alice to Charlie is 400 km, including 3 classical relays, and the distance from Bob to Charlie is 200 km, including 2 classical relays. Figure 3(b) shows the BB84-QKD architecture in which classical signals and quantum signals are simultaneously transmitted in MCF. The transmission distance from Alice to Bob is 600 km, spanning a total of 5 classical relays. In the simulation architecture, the distance of each segment is an example, and the architecture can be applied to any segment distance and total distance scenarios. If in other distance scenarios, the trend of the relationship between the noise power (or secure key rate) and distance is similar for each QKD protocol, the only difference is the position of the sudden change point of the noise power (or secure key rate) caused by node loss.

**Figure 4:**
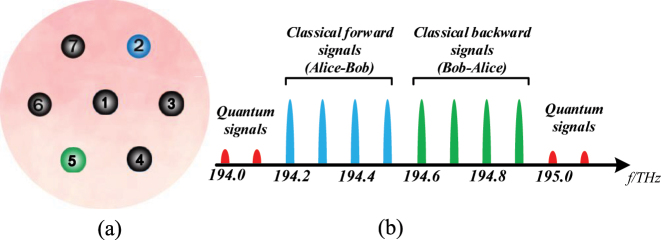
The core and wavelength assignments. (a) Core assignment in 7-core fiber (classical forward signals are transmitted in core 2, and classical backward signals are transmitted in core 5. Quantum signals are transmitted in the core 2 and core 5). (b) Wavelength assignment.

A 7-core fiber is used in the simulation, and the 7-core fiber cross-section is shown in Figure 4(a). To evaluate the feasibility of architectures and schemes, we consider the worst wavelength allocation. As shown in Figure 4(b), the forward classical signals have 4 frequencies, which are 194.2 THz, 194.3 THz, 194.4 THz, and 194.5 THz, and are transmitted in the core 2. The classical backward signals also have 4 frequencies, which are 194.6 THz, 194.7 THz, 194.8 THz, and 194.9 THz, and are transmitted in core 5. The quantum channels are 194.0 THz, 194.1 THz, 195.0 THz, and 195.1 THz, and allocated on core 2 and core 5.

**Figure 5:**
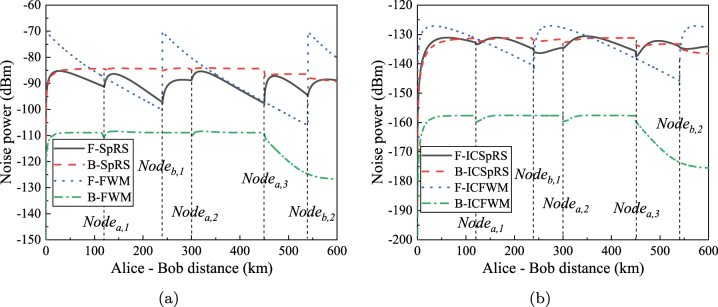
The relationship between noise power and transmission distance in the simulation architecture of asymmetric SNS-QKD (The power per classical signal is 0 dBm, and the quantum channel is 195.0 THz). (a) Intra-core noises. (b) Inter-core noises.

Figure 5 shows the relationship between different noise power and transmission distance in the simulation architecture of Figure 3(a). In the simulation, the power of classical signal per channel is 0 dBm. Figure 5(a) shows the intra-core noises. F-FWM noise power, B-FWM noise power, F-SpRS noise power, and B-SpRS noise power are calculated according to Equations (5), (9), (11) and (13), respectively. From Alice to Charlie, the quantum signals in core 2 are affected by the forward noises from the classical forward signals in core 2, and the quantum signals in core 5 are affected by the backward noises from the classical backward signals in core 5. From Bob to Charlie, the quantum signals in core 5 are affected by the forward noises from the classical forward signals in core 5, and the quantum signals in core 2 are affected by the backward noises from the classical forward signals in core 2.

There are 5 classical relays in total and the relationship between classical relays and transmission distance is corresponding. The transmission distance is the sum of the distance between Alice and Charlie and the distance between Bob and Charlie, where the ratio between two distances is fixed to be 2: 1. It passes through the first classical relay Node_*a*,1_ when the transmission distance reaches 120 km, and the distance between Alice and Charlie is 80 km, and the distance between Bob and Charlie is 40 km. When the transmission distance reaches 240 km, it will pass through the second classical relay Node_*b*,1_. The distance between Alice and Charlie is 160 km, and the distance between Bob and Charlie is 80 km. Therefore, the corresponding transmission distances of Node_*a*,2_, Node_*a*,3_, and Node_*b*,2_ can be obtained, which are 300 km, 450 km, and 540 km, respectively.

For F-FWM and B-FWM noises, FWM noise power is very weak with more than 200 GHz channel spacing. Therefore, they are only generated from the classical backward signals, and F-FWM only exists in Bob to Charlie, and B-FWM only exists in Alice to Charlie. Corresponding to the power of F-FWM noise only in Node_*b*,1_ and Node_*b*,2_ nodes have a sharply decreasing trend, and the power of B-FWM in Node_*a*,1_, Node_*a*,2_, and Node_*a*,3_ also have a decreasing trend. The FWM noise power is 0 mW at 0 km, which is not easy to observe because the *y*-axis is logarithmic. However, F-SpRS and B-SpRS noise will be generated at each node because of the wide spectrum of Raman scattering noise.

Figure 5(b) shows the power of the inter-core noises. F-ICFWM noise power, B-ICFWM noise power, F-ICSpRS noise power and B-ICSpRS noise power are calculated according to Equations (A.1)–(A.4), respectively. The correspondence between the power of inter-core noises and the node position is similar to the relationship between power of intra-core noises and node position. For F-ICFWM noise, the relationship with the transmission distance is different from that of F-FWM, and the peak of F-ICSpRS noise shifts back over the transmission distance. This is mainly because the generation of inter-core noise is the result of the cascade of intra-core noise and inter-core crosstalk noise, and they are all related to the transmission distance, so the position of the peak power is shifted back on each segment compared to the intra-core noise. The reason for the drop of B-FWM and B-ICFWM noises in the last segment is due to not utilizing the EDFA to amplify the classical signal when the classical signal passes through Charlie, but each of the previous segments will pass through the EDFA to amplify the classical signal.

**Figure 6:**
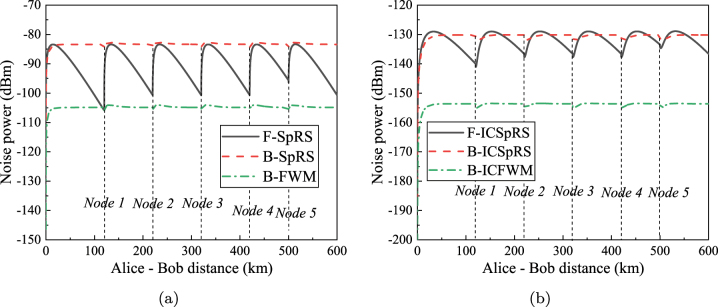
The relationship between noise power and transmission distance in the simulation architecture of BB84-QKD (The power per classical signal is 0 dBm, and the quantum channel is 195.0 THz). (a) Intra-core noises. (b) Inter-core noises.

Figure 6 shows the relationship between the power of various noises and transmission distance in BB84-QKD architecture. F-FWM noise power, B-FWM noise power, F-SpRS noise power, and B-SpRS noise power are calculated according to Equations (14)–(17), respectively. F-ICFWM noise power, B-ICFWM noise power, F-ICSpRS noise power, and B-ICSpRS noise power are calculated according to Equations (B.1)–(B.4), respectively. The quantum signals are affected by the forward noises from classical forward signals and the backward noises from classical backward signals. The signal power of each channel is 0 dBm. The channel spacing between forward classical signals and the quantum signals is far, about 500 GHz, so there is no F-FWM noise and F-ICFWM noise. This is different from asymmetric SNS-QKD. In asymmetric SNS-QKD, from Alice to Charlie, the classical signal and the quantum signal go in the same direction and from Bob to Charlie, the classical signal and the quantum signal go in the opposite direction. Therefore, both F-FWM and B-FWM noises exist in asymmetric SNS-QKD, but only B-FWM noise exists in BB84-QKD. Figure 6(a) shows the intra-core noises. The position of the classical relays and the transmission distance is also in one-to-one correspondence. The relationship between the inter-core noises and the transmission distance is shown in Figure 6(b), and each type of noise also exhibits similar periodic trends.

### 4.2 The impact of noises on secure key rate

**Figure 7:**
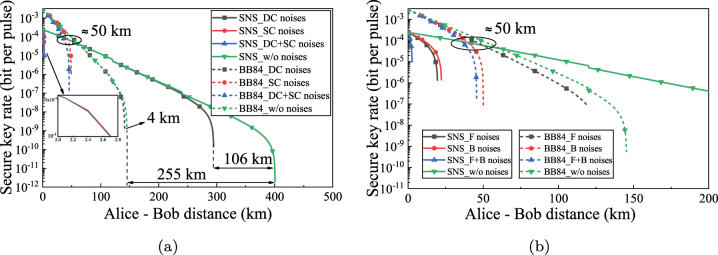
The relationship between secure key rate and transmission distance. (a) Under the influence of intra-core and inter-core noises. DC, Classical signals and quantum signals are allocated in different cores; SC, Classical signals and quantum signals are allocated in the same core. (b) Under the influence of forward and backward noises. F, forward; B, backward. (The power per classical signal is −20 dBm, and the quantum channel is 195.0 THz).

For the above-mentioned noises, we further study the impact on the performance of QKD system. Under the influence of intra-core noise, the maximum tolerable classical signal power of asymmetric SNS-QKD is −20 dBm, and BB84-QKD can exceed −10 dBm. In order to study under the condition that all QKD systems can work normally, we set the classical signal power per channel to −20 dBm in Figure 7 though it will degrade the performance of classical systems. In the simulation of BB84-QKD and asymmetric SNS-QKD, the length of the key is infinite. In BB84-QKD, the parameter setting is according to [54]. In the secure key rate calculation of the asymmetric SNS-QKD, we performed manual parameter optimization, the signal state intensity and decoy state intensities are required to satisfy constraints. The constraints need to be satisfied *μ*_*a*_ *η*_*a*_ = *μ*_*b*_ *η*_*b*_, *ω*_*a*_ *η*_*a*_ = *ω*_*b*_ *η*_*b*_, and *υ*_*a*_ *η*_*a*_ = *υ*_*b*_ *η*_*b*_. For other QKD parameters in Table 1, we refer to the current QKD experiments [51, 54], [55], [56], [57].

Figure 7(a) shows the influence of intra-core noises and inter-core noises on the QKD performance, which is studied in the asymmetric SNS-QKD architecture and BB84-QKD architecture respectively. The discontinuous points are caused by the loss introduced at the nodes. For the performance comparison of two architectures, in short-distance transmission, that is, the transmission distance is less than 50 km, the secure key rate of the BB84-QKD architecture is higher than that of the asymmetric SNS-QKD architecture. With the increase of the secure transmission distance, the secure key rate of BB84-QKD architecture is significantly reduced, and the secure key rate of the asymmetric SNS-QKD architecture is higher than that of the BB84-QKD architecture. For the comparison of the farthest secure transmission distance of each scheme, in the transmission without the coexistence of classical signals, SNS-QKD extends the secure transmission distance about 255 km compared to BB84-QKD, showing the excellent advantages of SNS-QKD in long-distance transmission. When quantum signals are transmitted with classical signals, the inter-core noises shorten the secure transmission distance about 106 km in SNS-QKD. However, the inter-core noises in BB84-QKD only shorten the secure transmission distance about 4 km. Furthermore, when the intra-core noises and inter-core noises exist, the maximum secure transmission distance of BB84-QKD exceeds that of SNS-QKD. This conclusion once again shows that the anti-noise ability of BB84-QKD is stronger than SNS-QKD, but in low-noise scenarios, SNS-QKD presents excellent advantages. In addition, we conclude that when the classical signals and quantum signals are transmitted in the same core, the BB84-QKD architecture is preferred. When the classical signals and quantum signals are transmitted in different cores, the asymmetric SNS-QKD architecture is preferred.

Figure 7(b) shows the relationship between the secure key rate and transmission distance under the interference of forward noises and backward noises. Similarly, we can also find that when the transmission distance is less than 50 km, the secure key rate of BB84-QKD is higher than that of SNS-QKD. When the transmission distance is longer than 50 km, the advantages of SNS-QKD can be presented. In SNS-QKD, forward noises and backward noises have heavier interference to system performance of QKD. Also, the effect of forward noises is more serious than that of backward noises. However, in the BB84-QKD, the backward noises are heavier than the forward noises. This is mainly because the BB84-QKD is not interfered by the forward FWM noise.

**Figure 8:**
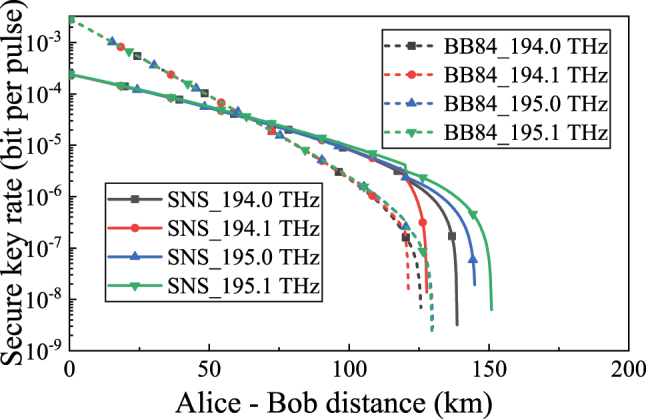
The relationship between secure key rate and transmission distance with different quantum channels (The power per classical signal is 0 dBm, and the quantum channel are 194.0 THz, 194.1 THz, 195.0 THz, and 195.1 THz).

In order to make the classical communication system meet the sensitivity requirements of the receiver, we set the classical signal power of each channel to 0 dBm for analysis. Figure 8 shows the performance of the asymmetric SNS-QKD and BB84-QKD systems on 4 quantum channels of 194.0 THz, 194.1 THz, 195.0 THz, and 195.1 THz when the classical signal and the quantum signal are transmitted in different cores. When the classical signal power per channel is 0 dBm, the transmission distance of asymmetric SNS-QKD can be extended more than that of BB84-QKD. Therefore, it can also be concluded that when the classical signal and the quantum signal are transmitted in different cores, the asymmetric SNS-QKD is preferred.

## 5 Experiments and results

**Figure 9:**
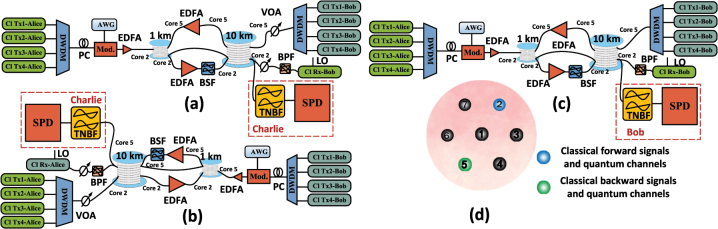
Experimental setup (Cl Tx-Alice/Cl Tx-Bob, The classical transmitter in Alice/Bob; Cl Rx-Alice/Cl Rx-Bob, the classical receiver in Alice/Bob; DWDM, dense wavelength division multiplexer; PC, polarization controller; AWG, arbitrary waveform generator; Mod., optical modulator; BPF, bandpass filter; EDFA, erbium doped fiber amplifier; BSF, bandstop filter; VOA, variable optical attenuation; LO, local oscillation; TNBF, tunable narrow bandpass filter; SPD, single photon detector). (a) Experimental measurement of Alice–Charlie in asymmetric SNS-QKD architecture. (b) Experimental measurement of Bob–Charlie in asymmetric SNS-QKD architecture. (c) Experimental measurement of Alice–Bob in BB84-QKD architecture. (d) Classical forward signals are transmitted in core 2, and classical backward signals are transmitted in core 5. Quantum channels are distributed in the core 2 and core 5.

Figure 9 shows the experimental setup. In order to use a 1 km 7-core fiber and a 10 km 7-core fiber to establish the transmission architecture of SNS-QKD when the classical amplifier exists, we first measure the noise of Alice–Charlie, and then take the measurement of Bob-Charlie, as shown in Figure 9(a) and (b). In Figure 9(a), the classical signal from Alice is quadrature phase shift keying (QPSK) modulated with a modulation rate 20 Gbps. After the modulated optical signal is amplified, it enters the core 2 of 1 km 7-core MCF. The classical signal from Bob is assigned to core 5, and the quantum channel is not in the same core, so it is simulated by continuous wave. The variable optical attenuation (VOA) is used to simulate the device attenuation of Bob–Charlie. For the 1 km and 10 km 7-core fibers, each core of the fibers is designed with a step index profile at a core pitch of 42.4 μm, the core diameter is 8.4 μm. The attenuation of fan-in and fan-out is between 2.9 dB and 4.2 dB. The bandstop filter (BSF) after erbium doped fiber amplifier (EDFA) is to filter the amplifier spontaneous emission noise. The bandpass filter (BPF) before the classical signal receiver is for receiving classical signals separately. The tunable narrow-band filter (TNBF) is to select the quantum channels, and the bandwidth is 0.12 nm. The single photon detector (SPD) can detect the noise photons on the quantum channels. The gate width is 2.1 ns, the efficiency is 15 %. Figure 9(c) experimentally evaluates the noise in the BB84-QKD architecture, and Figure 9(d) shows the core distribution.

Classical wavelengths of Alice–Bob are 194.2 THz, 194.3 THz, 194.4 THz, and 194.5 THz, and classical wavelengths of Bob–Alice are 194.6 THz, 194.7 THz, 194.8 THz and 194.9 THz. The power of each classical signal when entering the MCF is 0 dBm. The quantum channels are 194.0 THz, 194.1 THz, 195.0 THz, and 195.1 THz. Alice–Bob’s classical signal is distributed in core 2, Bob–Alice’s classical signal is distributed in core 5, and the quantum channel is distributed in core 2 and core 5.

**Figure 10:**
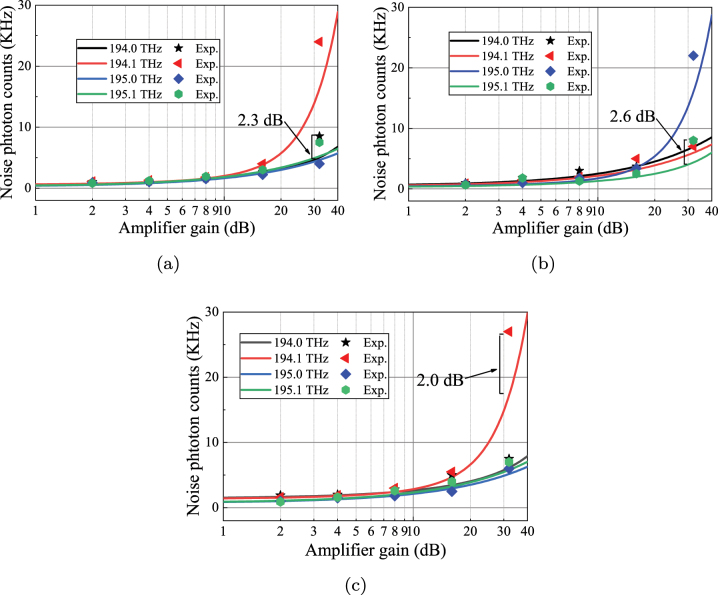
The relationship between noise photon counts and amplifier gain (Solid lines represent simulation results and dots are experimental results). The amplifiers represent two EDFAs between 1 km MCF and 10 km MCF, and they have the same gain. (a) Experimental measurement of Alice–Charlie in asymmetric SNS-QKD architecture. (b) Experimental measurement of Bob–Charlie in asymmetric SNS-QKD architecture. (c) Experimental measurement of Alice–Bob in BB84-QKD architecture.

In the experiment, the inter-core noise is weaker than the dark count, which is difficult to measure experimentally, so only the intra-core noise is measured. Figure 10(a) shows the intra-core noise photon counts of Alice–Charlie, when the amplifier gain is less than 10 dB, the noise photons on each quantum channel are close each other, and when the amplifier gain is greater than 10 dB, the noise photons on the quantum channel gradually increase, where noise on the quantum channel of 194.1 THz increases sharply due to the effect of forward intra-core FWM from the classical signals of Alice–Bob. The maximum difference between the simulation from the proposed theoretical model and experimental results is 2.3 dB. We believe that the error between the theoretical model and the experiment mainly includes the error introduced by the photon count fluctuation of the SPD itself. A similar trend also exists in Bob–Charlie’s noise measurements as shown in Figure 10(b). Due to the forward intra-core FWM noise from Bob–Alice, the noise influence on the quantum channel 195.0 THz is the most significant. The maximum difference between the simulation and experimental results is 2.6 dB. The same results can also be obtained in Figure 10(c). Therefore, the proposed theoretical model matches the experimental results well in asymmetric SNS-QKD and BB84-QKD.

## 6 Conclusions

In this paper, we propose simultaneous transmission architectures in which QKD and classical relays coexist, including asymmetric SNS-QKD and BB84-QKD. Also, the theoretical models of SpRS noise and FWM noise existing in the architecture are established, including intra-core noises, inter-core noises, forward noises, and backward noises. Then, the calculation models of secure key rate with asymmetric SNS-QKD when the classical signals exist are proposed. Finally, simulation and experiment are performed. Each kind of noise presents a different trend with the increase of the transmission distance, but sharply decreasing trend occurs when passing through the classical relays. The asymmetric SNS-QKD architecture extends the secure transmission distance compared to the BB84-QKD architecture when there is no noise. The proposed theoretical model matches the experimental results well. Furthermore, in pursuit of better QKD performance, the asymmetric SNS-QKD architecture is suitable for transmission of classical signals and quantum signals in different cores over MCF.

For a large-capacity transmission medium, in addition to MCF, the transmission of quantum signals and classical signals in new fibers such as ultra-low-loss fibers and hollow-core fibers have advantages in improving secure key rates and reducing fiber nonlinear effects, respectively. Meanwhile, in addition to reducing the noise on the quantum channels and adopting TF-QKD, there are other ways to extend the secure transmission distance, such as optimizing the parameters of the QKD system and reducing the link loss. These are important directions for future development and research about QKD.

## Appendix A: Theoretical model of inter-core noise in asymmetric SNS-QKD

F-ICFWM noise is generated by the interaction of intra-core FWM noise and ICXT noise when classical and quantum signals are transmitted in different cores and in the same direction. B-ICFWM noise is generated by the interaction of intra-core FWM noise, back Rayleigh scattering noise and ICXT noise when classical and quantum signals are transmitted in different cores and in opposite directions. F-ICSpRS and F-ICFWM are similar in principle, and B-ICSpRS noise and B-ICFWM are also similar in principle. The definitions and formula are given in Ref [58].

In order to simplify the calculation process, the calculation models of F-ICFWM noise, B-ICFWM noise, F-ICSpRS noise, and B-ICSpRS noise in the simultaneous transmission architecture are directly calculated here, as shown in Equations (A.1) to (A.4), respectively.

(A.1)
$$\begin{array}{ll} P_{F-ICFWM}^{a,q} & =\overset{N}{\underset{j=1}{\sum }}\left[P_{F-ICFWM}^{L_{a,j}}\right.\\ & \;\left.\times \exp\left(-\alpha_{q}\cdot \overset{N}{\underset{i=j+1}{\sum }}\left(L_{a,i}\right)\right)\cdot \overset{N}{\underset{i=j}{\prod }}A_{a,i}\right]. \end{array}$$

(A.2)
$$\begin{array}{ll} P_{B-ICFWM}^{a,q} & =\overset{N}{\underset{j=1}{\sum }}\left\{P_{B-ICFWM}^{L_{a,j}}\overset{N-1}{\underset{i=j}{\prod }}\right.\\ & \;\left.\times \left[A_{a,i}\cdot \exp\left(-\alpha_{q}\cdot L_{a,i+1}\right)\right]\right\}A_{a,N}. \end{array}$$

(A.3)
$$\begin{array}{ll} P_{F-ICSpRS}^{a,q} & =\overset{N}{\underset{j=1}{\sum }}\left[P_{F-ICSpRS}^{L_{a,j}}\right.\left.\;\exp\left(-\alpha_{q}\cdot \overset{N}{\underset{i=j+1}{\sum }}\left(L_{a,i}\right)\right)\cdot \overset{N}{\underset{i=j}{\prod }}A_{a,i}\right]. \end{array}$$

(A.4)
$$\begin{array}{ll} P_{B-ICSpRS}^{a,q} & =\overset{N}{\underset{j=1}{\sum }}\left\{P_{B-ICSpRS}^{L_{a,j}}\overset{N-1}{\underset{i=j}{\prod }}\right.\\ & \;\left.\times \left[A_{a,i}\cdot \exp\left(-\alpha_{q}\cdot L_{a,i+1}\right)\right]\right\}A_{a,N}. \end{array}$$

## Appendix B: Theoretical model of inter-core noise in BB84-QKD

For the simultaneous transmission architecture of BB84-QKD and classical signals shown in Figure 2, the calculation models of F-ICFWM noise, B-ICFWM noise, F-ICSpRS noise, and B-ICSpRS noise are directly calculated here, as shown in Equations (B.1) to (B.4), respectively.

(B1)
$$\begin{array}{ll} P_{F-ICFWM}^{q} & =\overset{N}{\underset{j=1}{\sum }}\left[P_{F-ICFWM}^{L_{j}}\right.\\ & \;\left.\times \exp\left(-\alpha_{q}\cdot \overset{N}{\underset{i=j+1}{\sum }}\left(L_{i}\right)\right)\cdot \overset{N}{\underset{i=j}{\prod }}A_{i}\right]. \end{array}$$

(B2)
$$\begin{array}{ll} P_{B-ICFWM}^{q} & =\overset{N}{\underset{j=1}{\sum }}\left\{P_{B-ICFWM}^{L_{j}}\overset{N-1}{\underset{i=j}{\prod }}\right.\\ & \;\left.\times \left[A_{i}\cdot \exp\left(-\alpha_{q}\cdot L_{i+1}\right)\right]\right\}A_{N}. \end{array}$$

(B3)
$$\begin{array}{ll} P_{F-ICSpRS}^{q} & =\overset{N}{\underset{j=1}{\sum }}\left[P_{F-ICSpRS}^{L_{j}}\exp\left(-\alpha_{q}\cdot \overset{N}{\underset{i=j+1}{\sum }}\left(L_{i}\right)\right)\right.\\ & \;\left.\cdot \overset{N}{\underset{i=j}{\prod }}A_{i}\right]. \end{array}$$

(B4)
$$\begin{array}{ll} P_{B-ICSpRS}^{q} & =\overset{N}{\underset{j=1}{\sum }}\left\{P_{B-ICSpRS}^{L_{j}}\overset{N-1}{\underset{i=j}{\prod }}\right.\\ & \;\left.\times \left[A_{i}\cdot \exp\left(-\alpha_{q}\cdot L_{i+1}\right)\right]\right\}A_{N}. \end{array}$$

## References

[1] Kaur K., Garg S., Kaddoum G., Bou-Harb E., Choo K. K. R. (2020). A big data-enabled consolidated framework for energy efficient software defined data centers in iot setups. IEEE Trans. Ind. Inf..

[2] Preskill J. (2018). Quantum computing in the nisq era and beyond. Quantum.

[3] Bennett C. H., Brassard G. (1984). Quantum cryptography: public key distribution and coin tossing. Proceedings of the IEEE International Conference on Computers, Systems and Signal Processing.

[4] Gisin N., Ribordy G., Tittel W., Zbinden H. (2002). Quantum cryptography. Rev. Mod. Phys..

[5] Lucamarini M., Yuan Z. L., Dynes J. F., Shields A. J. (2018). Overcoming the rate-distance limit of quantum key distribution without quantum repeaters. Nature.

[6] Ma X., Zeng P., Zhou H. (2018). Phase-matching quantum key distribution. Phys. Rev. X.

[7] Wang X. B., Yu Z. W., Hu X. L. (2018). Twin-field quantum key distribution with large misalignment error. Phys. Rev. A.

[8] Cui C., Yin Z. Q., Wang R. (2019). Twin-field quantum key distribution without phase postselection. Phys. Rev. Appl..

[9] Chen J. P., Zhang C., Liu Y. (2021). Twin-field quantum key distribution over a 511 km optical fibre linking two distant metropolitan areas. Nat. Photonics.

[10] Pittaluga M., Minder M., Lucamarini M. (2021). 600-km repeater-like quantum communications with dual-band stabilization. Nat. Photonics.

[11] Chen T. Y., Jiang X., Tang S. B. (2021). Implementation of a 46-node quantum metropolitan area network. NPJ Quantum Inf..

[12] Chen J. P., Zhang C., Liu Y. (2022). Quantum key distribution over 658 km fiber with distributed vibration sensing. Phys. Rev. Lett..

[13] Wang S., Yin Z. Q., He D. Y. (2022). Twin-field quantum key distribution over 830-km fibre. Nat. Photonics.

[14] Dynes J., Wonfor A., Tam W. S. (2019). Cambridge quantum network. NPJ Quantum Inf..

[15] Martin V., Aguado A., Brito J. (2019). Quantum aware sdn nodes in the madrid quantum network. 2019 21st International Conference on Transparent Optical Networks (ICTON).

[16] Eriksson T. A., Puttnam B. J., Rademacher G. (2019). Crosstalk impact on continuous variable quantum key distribution in multicore fiber transmission. IEEE Photonics Technol. Lett..

[17] Bacco D., Da Lio B., Cozzolino D. (2019). Boosting the secret key rate in a shared quantum and classical fibre communication system. Commun. Phys..

[18] Peters N., Toliver P., Chapuran T. (2009). Dense wavelength multiplexing of 1550 nm qkd with strong classical channels in reconfigurable networking environments. New J. Phys..

[19] Eraerds P., Walenta N., Legré M., Gisin N., Zbinden H. (2010). Quantum key distribution and 1 gbps data encryption over a single fibre. New J. Phys..

[20] Xavier G. B., de Faria G. V., da Silva T. F., Temporão G. P., von der Weid J. (2011). Active polarization control for quantum communication in long-distance optical fibers with shared telecom traffic. Microw. Opt. Technol. Lett..

[21] Fröhlich B., Lucamarini M., Dynes J. F. (2017). Long-distance quantum key distribution secure against coherent attacks. Optica.

[22] Dynes J. F., Tam W. W., Plews A. (2016). Ultra-high bandwidth quantum secured data transmission. Sci. Rep..

[23] Sun Y., Lu Y., Niu J., Ji Y. (2016). Reduction of fwm noise in wdm-based qkd systems using interleaved and unequally spaced channels. Chin. Opt Lett..

[24] Niu J. N., Sun Y. M., Cai C., Ji Y. F. (2018). Optimized channel allocation scheme for jointly reducing four-wave mixing and Raman scattering in the dwdm-qkd system. Appl. Opt..

[25] da Silva T. F., Xavier G. B., Temporão G. P., von der Weid J. P. (2014). Impact of Raman scattered noise from multiple telecom channels on fiber-optic quantum key distribution systems. J. Lightwave Technol..

[26] Bahrani S., Razavi M., Salehi J. A. (2018). Wavelength assignment in hybrid quantum-classical networks. Sci. Rep..

[27] Lin R., Chen J. (2021). Minimizing spontaneous Raman scattering noise for quantum key distribution in wdm networks. Optical Fiber Communication Conference (OFC), Conference Proceedings.

[28] Puttnam B. J., Luís R. S., Rademacher G. (2022). c-and l-band transmission over a 157 nm bandwidth using doped fiber and distributed Raman amplification. Opt. Express.

[29] Essiambre R. J., Kramer G., Winzer P. J., Foschini G. J., Goebel B. (2010). Capacity limits of optical fiber networks. J. Lightwave Technol..

[30] Randel S., Ryf R., Sierra A. (2011). 6× 56-gb/s mode-division multiplexed transmission over 33-km few-mode fiber enabled by 6× 6 mimo equalization. Opt. Express.

[31] Saitoh K., Matsuo S. (2013). Multicore fibers for large capacity transmission. Nanophotonics.

[32] Richardson D., Fini J., Nelson L. E. (2013). Space-division multiplexing in optical fibres. Nat. Photonics.

[33] Winzer P. J. (2014). Making spatial multiplexing a reality. Nat. Photonics.

[34] Saitoh K., Matsuo S. (2016). Multicore fiber technology. J. Lightwave Technol..

[35] Hayashi T. (2022). Multi-core fiber technology from design to deployment. 2022 European Conference on Optical Communication (ECOC).

[36] Hayashi T., Nagashima T., Morishima T., Saito Y., Nakanishi T. (2019). Multi-core fibers for data center applications. 45th European Conference on Optical Communication (ECOC 2019).

[37] Da Lio B., Cozzolino D., Biagi N. (2021). Path-encoded high-dimensional quantum communication over a 2-km multicore fiber. NPJ Quantum Inf..

[38] Da Lio B., Bacco D., Cozzolino D. (2019). Stable transmission of high-dimensional quantum states over a 2-km multicore fiber. IEEE J. Sel. Top. Quantum Electron..

[39] Hayashi T., Taru T., Shimakawa O., Sasaki T., Sasaoka E. (2011). Design and fabrication of ultra-low crosstalk and low-loss multi-core fiber. Opt. Express.

[40] Kong W., Sun Y., Cai C., Ji Y. (2020). Impact of modulation formats and bandwidth on quantum secured 5g optical fronthaul over multicore fiber. CLEO: Applications and Technology.

[41] Lin R., Gan L., Udalcovs A. (2019). Spontaneous Raman scattering effects in multicore fibers: impact on coexistence of quantum and classical channels. Optical Fiber Communication Conference.

[42] Cai C., Sun Y., Ji Y. (2020). Intercore spontaneous Raman scattering impact on quantum key distribution in multicore fiber. New J. Phys..

[43] Kong W., Sun Y., Cai C., Ji Y. (2021). Impact of classical modulation signals on quantum key distribution over multicore fiber. J. Lightwave Technol..

[44] Kong W., Sun Y., Gao Y., Ji Y. (2022). Core and wavelength allocation of sending-or-not-sending quantum key distribution for future metropolitan networks over multicore fiber. Optical Fiber Communication Conference (OFC), Conference Proceedings.

[45] Dynes J., Kindness S., Tam S. B. (2016). Quantum key distribution over multicore fiber. Opt. Express.

[46] Cai C., Sun Y., Zhang Y., Zhang P., Niu J., Ji Y. (2019). Experimental wavelength-space division multiplexing of quantum key distribution with classical optical communication over multicore fiber. Opt. Express.

[47] Hugues-Salas E., Alia O., Wang R. (2020). 11.2 tb/s classical channel coexistence with dv-qkd over a 7-core multicore fiber. J. Lightwave Technol..

[48] Geng J., Fan-Yuan G. J., Li K. J. (2022). Integration in c-band between quantum key distribution and classical channel of 25 dbm launch power over multicore fiber media. *Opt. Lett.*.

[49] Suzuki K. I., Fujiwara M., Taguchi K. (2011). 128× 8 split and 80 km long-reach dual-rate 10g-epon transmission using alc hybrid burst-mode optical fiber amplifier and soa pre-amplifier. European Conference and Exposition on Optical Communications.

[50] Cai C., Sun Y., Ji Y. (2021). Simultaneous long-distance transmission of discrete-variable quantum key distribution and classical optical communication. IEEE Trans. Commun..

[51] Zhou X. Y., Zhang C. H., Zhang C. M., Wang Q. (2019). Asymmetric sending or not sending twin-field quantum key distribution in practice. Phys. Rev. A.

[52] Kawahara H., Medhipour A., Inoue K. (2011). Effect of spontaneous Raman scattering on quantum channel wavelength-multiplexed with classical channel. Opt. Commun..

[53] Lo H. K., Ma X., Chen K. (2005). Decoy state quantum key distribution. Phys. Rev. Lett..

[54] Wang B. X., Mao Y., Shen L. (2020). Long-distance transmission of quantum key distribution coexisting with classical optical communication over a weakly-coupled few-mode fiber. Opt. Express.

[55] Minder M., Pittaluga M., Roberts G. L. (2019). Experimental quantum key distribution beyond the repeaterless secret key capacity. Nat. Photonics.

[56] Geng J. Q., Fan-Yuan G. J., Wang S. (2021). Coexistence of quantum key distribution and optical transport network based on standard single-mode fiber at high launch power. Opt. Lett..

[57] Chen J. P., Zhang C., Liu Y. (2020). Sending-or-not-sending with independent lasers: secure twin-field quantum key distribution over 509 km. Phys. Rev. Lett..

[58] Kong W., Sun Y., Gao Y., Ji Y. (2022). Core and wavelength allocation schemes for noise suppression in quantum key distribution over multicore fiber. IEEE J. Sel. Top. Quantum Electron..

